# Ovarian hyperstimulation syndrome: severity predictors

**DOI:** 10.5935/1518-0557.20260034

**Published:** 2026

**Authors:** Magdalena Piróg, Olga Kacalska-Janssen, Alicja Ogiegło, Robert Jach

**Affiliations:** 1 Gynecological Endocrinology Department, Jagiellonian University Medical College, Krakow, Poland

**Keywords:** albumin, estradiol, hematocrit, infertility, ovarian hyperstimulation syndrome

## Abstract

**Objective:**

To evaluate clinical and laboratory parameters associated with the severity of ovarian hyperstimulation syndrome (OHSS) and to assess pregnancy outcomes in women hospitalized with moderate or severe OHSS. Several risk factors, including elevated antral follicle count (AFC), anti-Mullerian hormone (AMH), and serum estradiol (E2) levels, increase the risk of OHSS.

**Methods:**

We studied 71 women with a median age of 32.7 years and a body mass index (BMI) of 22.8 kg/m2 with moderate (n=38) and severe (n=31) OHSS. We measured E2, hemoglobin (Hb), hematocrit (Hct), creatinine, albumin (Alb), total protein (TP), D-dimer (DD), and alanine aminotransferase (ALT) levels on the day of hospitalization and every second day until discharge. Data on the length of hospitalization, along with pregnancy outcomes, were recorded.

**Results:**

There were no intergroup differences between women with moderate and severe OHSS in terms of age and BMI. The total pregnancy rate in both moderate (18/38) and severe (9/31) OHSS was 27/69; however, in women with severe OHSS, the positive pregnancy outcome was approximately half that observed in the moderate OHSS group and was accompanied by longer hospitalization (2.9-fold). All women with OHSS had elevated initial E2 levels (>6910pg/mL). Women with severe OHSS had higher initial E2 (*p*=0.001) and Hct (*p*=0.006) levels, together with lower Alb and TP concentrations (both *p*<0.001), compared with those with moderate OHSS.

**Conclusion:**

Elevated estradiol concentration is an associated factor for OHSS in women undergoing COH. Basal serum albumin concentration, together with total protein level and hematocrit, are predictive factors for OHSS severity.

## INTRODUCTION

Ovarian hyperstimulation syndrome (OHSS) is a complication of controlled ovarian stimulation (COH) in women undergoing assisted reproductive technologies (ART), occurring in up to 30% of cycles (Nelson, 2017; Timmons *et al*., 2019). OHSS is characterized by bilateral enlargement of highly luteinized ovaries (Ovarian Stimulation TEGGO *et al*., 2020). Secondary complications related to arteriolar vasodilatation, increased capillary permeability, and a prothrombotic effect are responsible for clinical features such as nausea, vomiting, mild to severe abdominal pain/distension due to ascites and increased ovarian size, shortness of breath due to pleural effusion, and reduced urine output (Romito *et al*., 2017; Schon *et al*., 2022).

Currently, there is no consensus on the classification of OHSS, although in 2016 the Royal College of Obstetricians and Gynaecologists (RCOG) proposed a classification system based on symptoms and severity, in which OHSS was divided into mild (ovarian size usually <8 cm), moderate (ovarian size 8-12 cm), severe (ovarian size >12 cm), and critical (The Royal College of Obstetricians and Gynaecologists, 2016). Moderate OHSS clinical manifestations include abdominal pain, nausea, and vomiting, as well as ultrasound evidence of ascites. Severe OHSS may be associated with oliguria, unfavorable changes in sodium and potassium levels, and clinical ascites accompanied by hydrothorax. Critical OHSS may be related to life-threatening complications such as hepatorenal failure, acute respiratory distress syndrome, hemorrhage from ovarian rupture, thromboembolism, and even death. The prevalence of mild OHSS is underestimated, mostly because the symptoms may go unnoticed, whereas moderate-to-severe OHSS occurs in 1%-5% of COH (Sun *et al*., 2020).

Certain factors that play an important role in OHSS development, such as younger age, low body mass index (BMI), polycystic ovarian syndrome (PCOS), elevated antral follicle count (AFC), anti-Mullerian hormone (AMH), history of OHSS, and medication used for COH, including higher doses of gonadotropins and the use of human chorionic gonadotropin (hCG) as a trigger, together with an elevated serum estradiol (E2) level at the end of COH, have been identified (Timmons *et al*., 2019; Corbett *et al*., 2014).

Nevertheless, the above indicators do not provide any further information on the severity of OHSS. The prevention of OHSS, as well as limiting its development, has become more important with the rapid expansion of ART. Therefore, the aim of this study was to identify biochemical predictors of OHSS severity in hospitalized women.

## MATERIALS AND METHODS

### Study population

**Figure 1:**
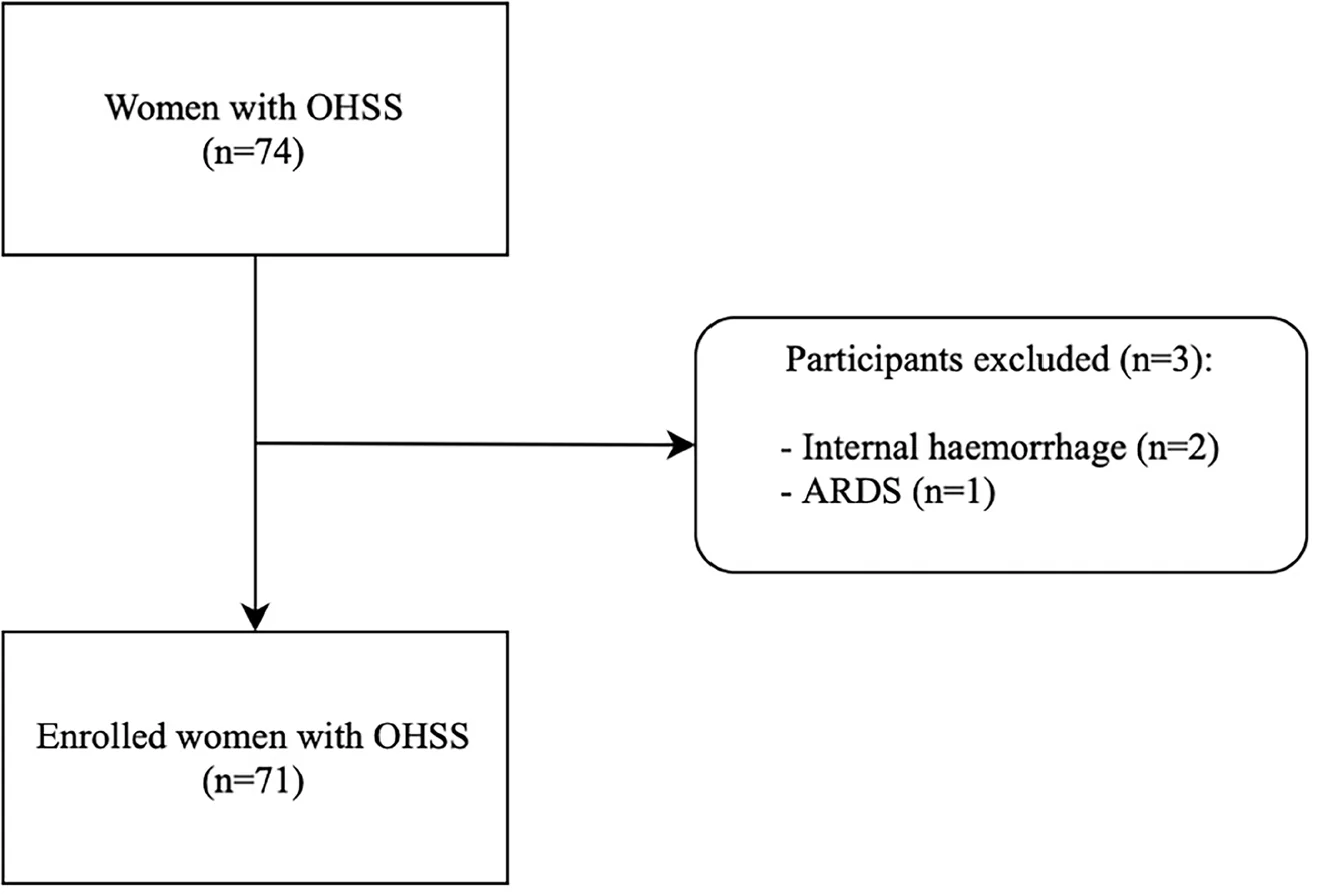
The study flowchart of women with ovarian hyperstimulation syndrome (OHSS) enrolled to the study. ARDS, acute respiratory distress syndrome

In this retrospective study, records of women with OHSS referred to the Department of Gynecological Endocrinology between January 2019 and May 2024 were reviewed. All women enrolled in this research fulfilled at least one of the following criteria: 1) OHSS after COH for in vitro fertilization (IVF), 2) abdominal pain, and 3) enlarged ovaries. Exclusion criteria were as shown in the study flowchart (Figure 1). We collected demographic data, information on risk factors, and comorbidities.

### Laboratory investigations

Venous blood samples were drawn between 08:00 and 10:00 AM after an overnight fast. Blood cell count, total protein (TP), albumin (Alb), creatinine, alanine aminotransferase (ALT), estradiol (E2), and D-dimer (D-D) were assayed by routine laboratory techniques. All laboratory investigations were performed in the University Hospital Laboratory using standardized assays.

### OHSS diagnosis

The diagnosis of OHSS was based on the RCOG criteria as well as OHSS stage stratification (The Royal College of Obstetricians and Gynaecologists, 2016). Mild OHSS was diagnosed when abdominal bloating, mild abdominal pain, and an ovarian diameter <8 cm were confirmed. Moderate abdominal pain accompanied by nausea +/- vomiting, ultrasound evidence of ascites, and ovarian enlargement of 8-12 cm were confirmed in women with moderate disease. Clinical ascites or pleural effusion, oliguria (<300 ml/d or <30 ml/h), hematocrit (Hct) >0.45, hyponatremia (sodium <135 mmol/L), hypo-osmotic pressure (<282 mmol/L), hyperkalemia (potassium >5 mmol/L), hypoalbuminemia (serum albumin <35 g/L), and ovarian size >12 cm indicated severe OHSS. Critical OHSS may be related to life-threatening complications such as hepatorenal failure, acute respiratory distress syndrome, hemorrhage from ovarian rupture, together with thromboembolism.

The clinical examination of OHSS included a transvaginal ultrasound scan (TUS) to measure ovarian volume and free fluid in the Douglas pouch, a transabdominal ultrasound scan to confirm ascites, chest X-ray or chest ultrasound scan to confirm hydrothorax and/or pericardial effusion, and measurement of body weight and abdominal circumference.

Clinical parameters related to OHSS were evaluated on admission to the hospital (Day 1), every second day during hospitalization, and at the end of hospitalization (Day ex). Data regarding treatment, such as low-molecular-weight heparin, albumin infusion, furosemide, and cabergoline administration, were recorded. We collected data including the time from oocyte pick-up (OPU), embryo transfer (ET), and length of hospitalization.

### OHSS treatment

All women, regardless of the type of OHSS - moderate or severe - received intravenous infusion of albumin and furosemide (for 5 days) and continued cabergoline for 8 days from the day of the trigger, which had been prescribed by the doctor at the infertility clinic. Moreover, all participants received thromboprophylaxis according to the guidelines (Piróg *et al*., 2016).

### Compliance with ethical standards

The Ethics Committee at Jagiellonian University Medical College approved the study, and the participants provided informed consent in accordance with the Declaration of Helsinki.

### Statistical analysis

Categorical variables are presented as numbers and percentages, and continuous variables are expressed as mean +/- standard deviation or median and interquartile range (IQR), as appropriate. The Kolmogorov-Smirnov test was used to assess conformity with a normal distribution, whereas non-normally distributed data were analyzed using the Kruskal-Wallis test followed by the Mann-Whitney comparison test. Categorical variables were analyzed using either the chi-square test or Fisher’s exact test. Pearson’s correlation coefficient (Pearson’s r) or Spearman’s rank correlation coefficient was calculated to assess linear correlations between variables with a normal or non-normal distribution, respectively. Associations between the variables were expressed as odds ratios with 95% confidence intervals. Two-sided *p*-values <0.05 were considered statistically significant. Statistical analysis was performed using STATISTICA 13.0 software (StatSoft, Poland).

## RESULTS

The final analysis included 71 women with mild (n=2, 2.8%), moderate (n=38, 53.5%), and severe (n=31, 43.5%) OHSS (Table 1). There were no intergroup differences between women with moderate and severe OHSS regarding age and BMI. The causes of infertility were comparable between moderate and severe OHSS, except for slightly higher tubal pathology and a lower male factor in the severe OHSS group (Table 1). There were no differences in the time from OPU to the day of admission to the hospital or in the number of ETs between the moderate and severe OHSS groups. More women in the severe OHSS group underwent COH with an agonist protocol compared with the moderate group. Changes in ovarian size and the presence of free fluid in women with different severity of OHSS are described in Table 1. Women with severe OHSS received albumin infusion together with furosemide more frequently than women with moderate OHSS.

**Table 1:** Basic characteristics of women with ovarian hyperstimulation syndrome (OHSS).

	All (n=71)	Moderate OHSS (n=38)	Severe OHSS (n=31)	*p*
Baseline characteristic Age, yrs BMI, kg/m^2^	32.7±4.1 22.8±5.0	33.0±3.9 21.2±3.6	32.3±4.4 24.6±5.8	0.44 0.07
Concomitant diseases, n -Not related to infertility, n DM Hypothyroidism AH Varicose veins -Infertility cause, n PCOS Tubal Male Endometriosis Unexplained	3 7 2 1 20 15 15 16 3	2 4 1 1 11 7 10 8 2	1 3 1 0 9 8 5 8 1	- - - - - - - - -
Time from OPU, days	4.6±0.9	4.5±0.9	4.8±0.8	0.17
ET, n	44	24	20	0.14
Protocol type Agonist Antagonist	40 29	18 (43.4) 20 (52.6)	22 (71.0) 9 (29.0)	0.047
Ovarian size, mm Right Left	100 [86-130] 80 [73.4-99.4]	88.3 [83.5-97.8] 76.0 [69.5-80.0]	131 [122.0-144.5] 99.8 [81.0-120.0]	<0.001 <0.001
Ultrasound ascites, n (%)		24 (63.2)	4 (12.9)	-
Clinical ascites, n (%)	-	0 (0)	23 (74.2)	-
Pleural effusion, n (%)	-	0 (0)	14 (45.2)	-
Pericardial effusion, n (%)	-	0 (0)	3 (9.7)	-
Treatment Cabergoline, n Albumin, n Furosemide, n	59 21 30	33 7 10	26 14 20	0.31 0.03 0.03

Values are given as mean ± SD, median (interquartile range), or percentage.

Abbreviations: BMI, body mass index; OHSS, ovarian hyperstimulation syndrome; OPU, oocyte pick-up; ET, embryo transfer; DM, diabetes mellitus; AH, arterial hypertension; PCOS, polycystic ovary syndrome.

The total pregnancy rate in both moderate (18/38) and severe (9/31) OHSS was 27/69; however, in women with severe OHSS, the positive pregnancy outcome was approximately half that observed in the moderate OHSS group and was accompanied by longer hospitalization (2.9-fold) (Table 2).

**Table 2:** Differences in pregnancy rate, duration of hospitalization and basic laboratory parameters between women with moderate and severe OHSS.

	Moderate OHSS (n=38)	Severe OHSS (n=31)	*p*
Pregnancy rate, n	18	9	0.02
Duration of hospitalization, days	5.4±1.6	15.5±6.1	<0.001
Basic laboratory parameters			
Hb 1, g/dL	13.2 [11.9-14.2]	15.6 [13.7-16.7]	0.006
Hb max, g/dL	13.2 [12.2-14.4]	16.0 [14.2-16.7]	<0.001
Hb ex, g/dL	12.5 [11.4-13.1]	11.6 [10.8-12.7]	0.08
Hct 1, %	38.3 [34.9-40.7]	44.6 [39.8-46.5]	0.006
Hct max, %	38.9 [36.2-40.7]	45.2 [40.4-46.9]	<0.001
Hct ex, %	36.8 [33.5-38.3]	33.5 [32.2-36.5]	0.02
ALT 1, IU/L	16.0 [10.0-26.0]	20.0 [16.0-38.8]	0.21
ALT max, IU/L	20.0 [16.0-38.8]	77.0 [38.5-129.0]	0.03
ALT ex, IU/L	20.0 [16.0-35.0]	57.0 [30.3-87.3]	0.004
Alb 1, g/L	41.5 [39.0-45.0]	34.4 [31.8-36.2]	<0.001
Alb ex, g/L	42.0 [40.4-44.4]	44.8 [42.1-47.8]	<0.001
TP 1, g/L	67.3 [62.3-71.0]	58.0 [54.3-61.1]	<0.001
TP min, g/L	64.2 [60.6-67.1]	52.8 [50-58.1]	<0.001
TP ex, g/L	67.9 [65.7-71.0]	69.0 [64.2-71.7]	0.97
Creatinine 1, µmol/L	61.5 [57.1-66.6]	75.0 [64.6-81.0]	0.003
Creatinine max, µmol/L	65.3 [61.0-69.6]	77.9 [72.5-85.1]	0.001
Creatinine ex, µmol/L	60.0 [55.0-63.7]	62.8 [53.5-71.0]	0.26
D-D 1, µg/L	1.1 [0.7-1.6]	1.8 [1.4-2.2]	0.63
D-D max, µg/L	1.7 [1.0-4.2]	3.1 [2.0-6.2]	0.40
D-D ex, µg/L	2.1 [1.0-4.0]	3.0 [1.7-3.9]	0.50
E2 1, pg/mL	6910.0 [3893.0-8975.0]	16758.0 [10810.0-24687.0]	0.001
E2 max, pg/mL	8689.5 [4619.0-10039.0]	19070.0 [11229.0-29416.0]	<0.001
E2 ex, pg/mL	2887.0 [355.5-5402.5]	4985.5 [962.6-11566.8]	0.02

Values are given as mean ± SD, median (interquartile range), or percentage.

Abbreviations: Hb, hemoglobin; Hct, hematocrit; ALT, alanine aminotransferase; Alb, albumin; TP, total protein; D-D, D-dimer; E2, estradiol; 1, day 1 (admission to the hospital); min/max, minimal/maximal value; ex, end of hospitalization.

**Figure 2:**
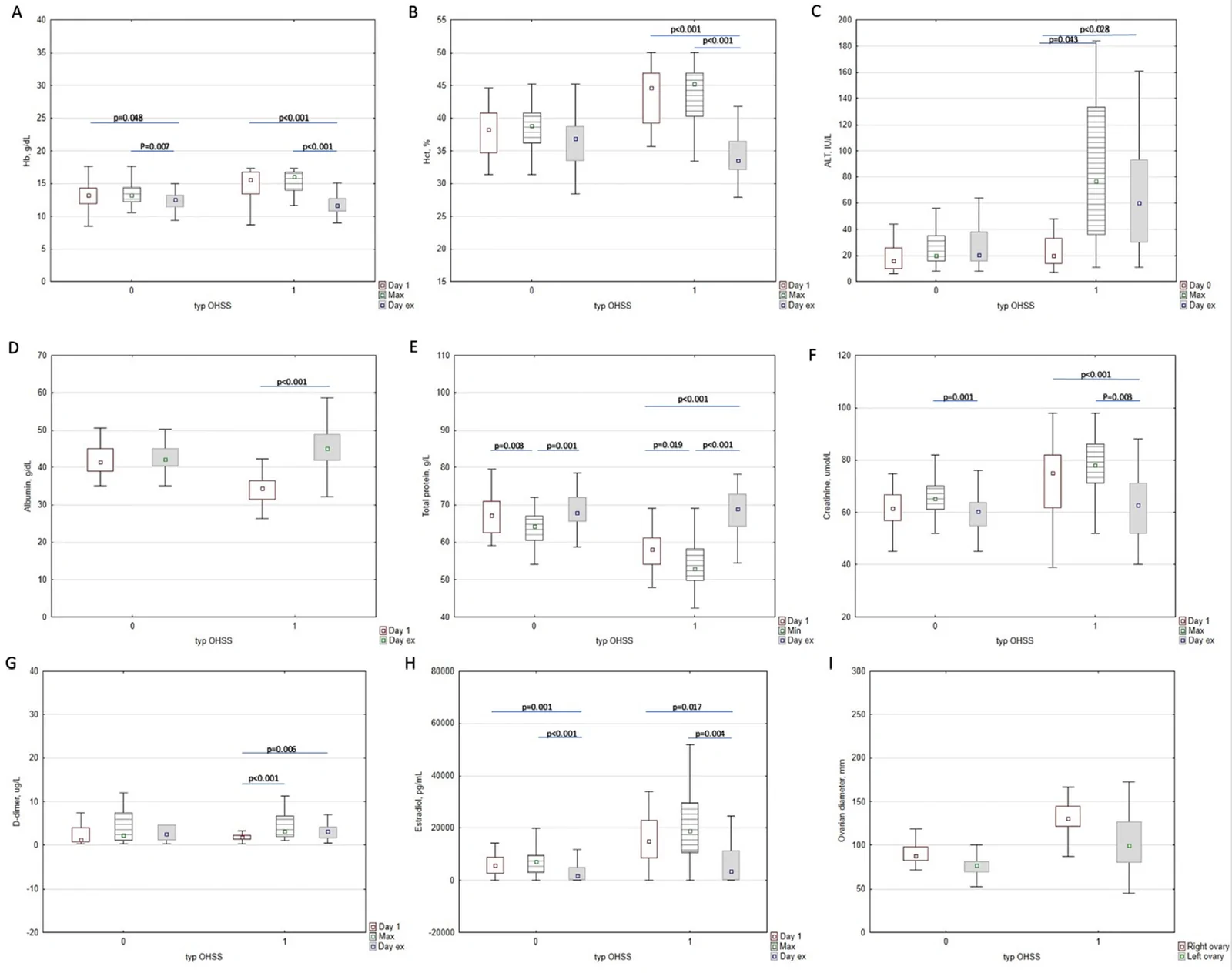
Changes in OHSS-related parameters (panels A-I) in women with moderate (0) and severe (1) OHSS on admission to the hospital (Day 1), on the day with minimal (min) or maximal (max) level, and at the end of hospitalization (Day ex).

Regarding hemoglobin (Hb) levels, initial and maximum Hb concentrations were higher in severe OHSS compared with moderate OHSS (+18.2% and +21.2%, respectively) (Table 2). At discharge, Hb levels were lower than the initial and maximum levels in moderate OHSS (both -5.3%) and severe OHSS (-25.6% and -27.5%, respectively) (Figure 2, panel A).

Women with severe OHSS had higher initial (+16.4%) and maximal (+16.2%) hematocrit (Hct) levels, along with lower discharge values (-9.0%), when compared with moderate OHSS. Severe OHSS was characterized by higher initial and maximum Hct levels in contrast to discharge values (33.1% and 34.3%, respectively; Figure 2, panel B). Severe OHSS was characterized by higher maximum and discharge ALT levels (approximately 3.9-fold and 2.9-fold, respectively) in contrast to moderate OHSS. Women with severe OHSS had lower initial ALT levels in contrast to maximum and discharge levels (approximately 3.9-fold and 2.9-fold, respectively; Figure 2, panel C).

Regarding Alb and TP, both levels were lower in women with severe versus moderate OHSS (-17.1% and -13.8%, respectively). Women with severe OHSS had a lower initial Alb level (-23.2%) when compared with the discharge Alb level (Figure 2, panel D). Intergroup analysis revealed a lower minimum TP level in contrast to both Day 1 and discharge levels in both the moderate (-4.6% and -5.4%, respectively) and severe groups (-9.0% and -23.5%, respectively). In the severe OHSS group, the initial Alb level was 15.9% lower than the discharge Alb level (Figure 2, panel E).

Initial and maximum creatinine levels were higher (+22% and +19.3%, respectively) in severe OHSS, in contrast to moderate OHSS. In both the moderate and severe OHSS groups, maximum creatinine levels were higher than discharge levels (+8.8% and +24.0%, respectively), and, additionally, a higher Day 1 creatinine level (+19.4%) was observed compared with the discharge level (Figure 2, panel F). Initial D-D levels were lower than the maximum and discharge levels (+72.2% and +66.7%, respectively) (Figure 2, panel G).

Regarding estradiol levels, women with severe OHSS had higher initial (2.4-fold), maximal (2.2-fold), and discharge levels (+72.7%) compared with the moderate OHSS group (Table 2). In both moderate and severe OHSS groups, maximum E2 levels were higher than the initial (2.4-fold and 3.4-fold, respectively) and discharge levels (3.0-fold and 3.8-fold, respectively) (Figure 2, panel H).

**Figure 3:**
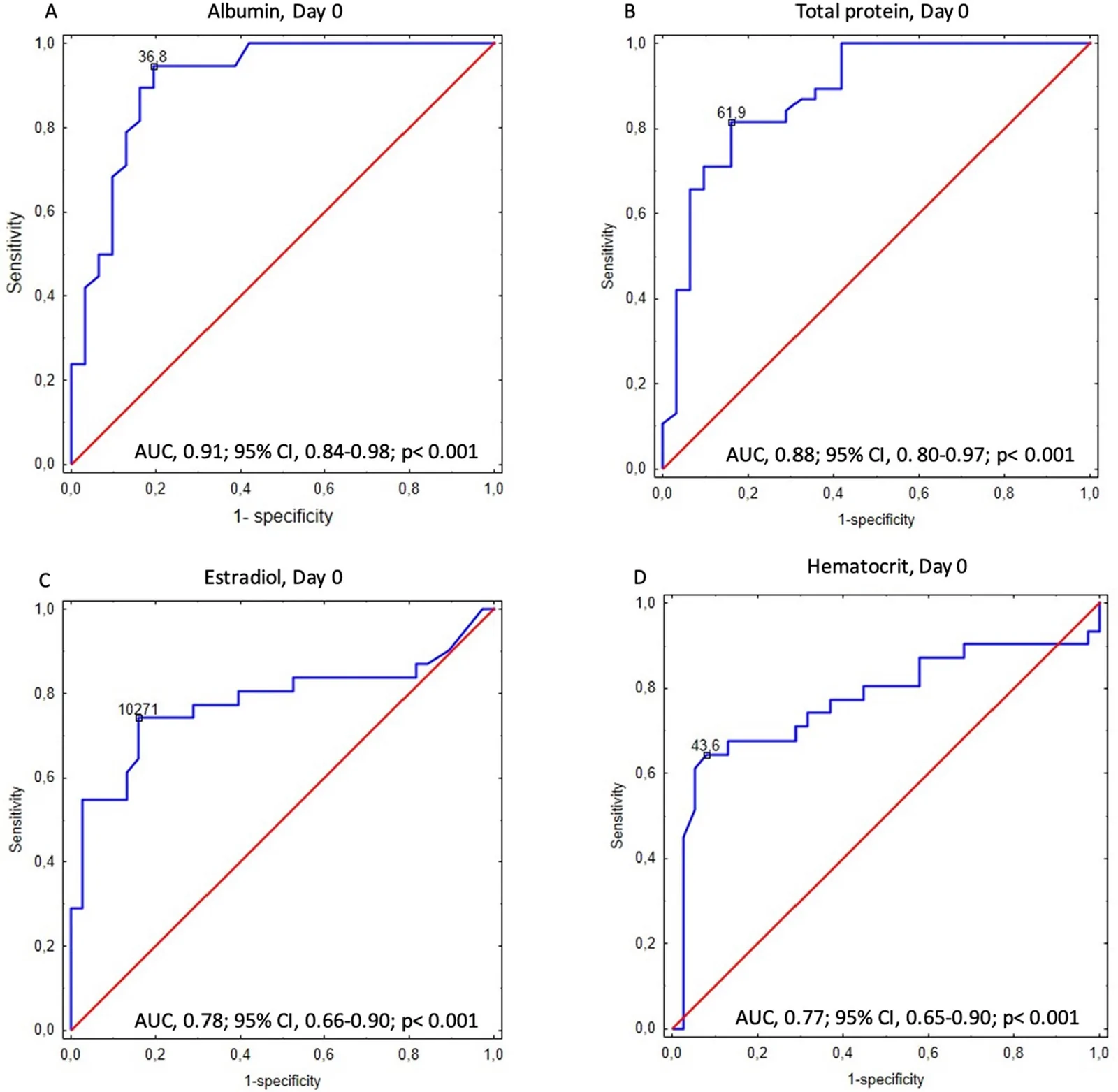
ROC curves for albumin Day 0 (panel A), total protein day 0 (panel B), estradiol Day 0 (panel C), hematocrit Day 0 (panel D). The outcomes investigated in the ROC analysis were moderate (panel A and B) along with severe OHSS occurrence (panel C and D).

We observed high accuracy for albumin and total protein in the prediction of mild OHSS (Figure 3, panels A and B, respectively), together with moderate accuracy for estradiol and hematocrit in the prediction of severe OHSS (Figure 3, panels C and D, respectively). Women with severe OHSS showed associations between estradiol and Hb (r=0.53, *p*=0.03), Hct (r=0.41, *p*=0.03), and Alb levels (r=-0.31, *p*=0.01).

## DISCUSSION

Our study is the first to show that decreased albumin and total protein levels, together with not only higher estradiol but also increased hematocrit levels, may help predict OHSS severity. The current findings provide new insight into possible mechanisms underlying the development of more advanced OHSS stages.

Given the increasing number of ART cycles over recent years, the prevention of OHSS has become even more important. The prediction of OHSS occurrence, especially the late form, is difficult; therefore, implementation of specific strategies is needed to minimize the prevalence of OHSS (Medwin *et al*., 2023).

Across predictive models for OHSS based on ovarian reserve markers and demographic factors, high AMH levels (≥3.5-5.0 ng/mL) and/or elevated AFC (≥20-24 follicles) are strongly associated with an enhanced response to COH, particularly in younger women (below 30 years) and those with a lower BMI (<22-23 kg/m2) (Ovarian Stimulation TEGGO *et al*., 2020). Several multivariable models including the above parameters have been proposed to stratify women into low-, intermediate-, and high-risk groups, enabling individualized stimulation strategies (Schouten *et al*., 2025).

The use of GnRH antagonist protocols, in contrast to agonist protocols, should be considered (Patel & Kashyap, 2024; Piróg *et al*., 2022). However, in our study, 40.8% of women who developed either moderate or severe OHSS were managed with the GnRH antagonist protocol. Moreover, another study comparing agonist and antagonist protocols showed that the antagonist group had a lower total recombinant follicle-stimulating hormone dose and a lower number of stimulation days, together with fewer oocytes retrieved. Despite similar fertilization rates, the implantation rate was lower, and the ongoing pregnancy rate was 20.3% versus 25.7% in the agonist group. Nevertheless, in the antagonist group, the incidence of OHSS was 2.4% (Borm & Mannaerts, 2000).

An additional safe approach in COH is the use of a GnRH agonist (GnRHa) trigger instead of hCG. In our study, all patients received hCG. Nevertheless, a few studies have shown that clinical pregnancy rates, together with miscarriage rates, were lower after embryo transfer following agonist trigger (Toftager *et al*., 2016; Xiao *et al*., 2014). Possible explanations include inadequate development of the corpora lutea or inadequate luteal support with estradiol and progesterone supplementation (Gonadotrophin-releasing hormone antagonists for assisted reproductive technology, 2011). Moreover, dopamine agonists, such as cabergoline, have been shown to reduce the incidence and severity of OHSS by decreasing vascular endothelial growth factor (VEGF)-mediated vascular permeability and, therefore, limiting third-space fluid accumulation (Tang *et al*., 2021). The combination of dopamine agonist administration with individualized luteal phase adjustments offers a safe and effective preventive approach, particularly in patients identified as being at high risk for OHSS.

The risk of OHSS development after COH is greater in women who achieve conception (Sun *et al*., 2020). In our study, 45% of women with severe OHSS and 75% of women with moderate OHSS had confirmed pregnancy. A possible strategy to decrease the risk of OHSS is the ‘freeze-all’ approach following oocyte retrieval, in which all oocytes/embryos are cryopreserved and subsequently transferred in a non-stimulated cycle (Yan *et al*., 2025; Wang *et al*., 2024). However, despite the so-called safe approaches provided by vitrification and subsequent transfer, there is still a chance of OHSS development (Iorio *et al*., 2022). Gurbuz *et al*. (2014) reported three cases of severe OHSS after the use of a GnRHa trigger and cycle segmentation, without either endogenous or exogenous hCG influence. Moreover, Fatemi *et al*. (2014) reported severe OHSS requiring hospitalization in two women who underwent vitrification of all embryos followed by a subsequent transfer.

The presence of OHSS is almost always related to increased estradiol levels. The current literature indicates that estradiol values above 3,500 pg/mL are particularly associated with an increased risk of OHSS in patients undergoing fertility treatment (Practice Committee of the American Society for Reproductive Medicine, 2016). In our study, women with OHSS, regardless of its severity, had E2 concentrations above 6910 pg/mL, and women with severe OHSS had higher E2 levels both at the beginning and at the end of hospitalization.

In our study, both initial TP and Alb levels were below the reference ranges in severe OHSS. The mechanism underlying the potential effect of intravenous fluids on OHSS has not been well investigated. One theory highlights a beneficial effect of the binding properties of albumin in neutralizing vascular permeability mediators that could be responsible for the onset of OHSS (Youssef *et al*., 2011). Another theory emphasizes that an appropriate albumin level promotes a rapid increase in intravascular volume and, therefore, prevents hypovolemia and hemoconcentration (McClelland, 1990). Taking into account that a single dose of hCG reaches a maximum blood concentration in about 12 h and is eliminated from the circulation after 5-6 days (Shah *et al*., 2014), the administration of intravenous fluid expanders immediately after oocyte retrieval might help with the excretion of hCG during the hours of peak concentration (Youssef & Mourad, 2016) and decrease the risk or severity of OHSS.

Our study has several limitations. First, it is a single-center retrospective study; however, it is hypothesis-generating. Nevertheless, the presented associations do not necessarily imply a cause-effect relationship; therefore, our results should be interpreted with caution. Second, in the present study, data regarding risk factors for OHSS before COH (i.e., AFC, AMH), as well as parameters assessed during COH (i.e., E2 concentration on the day of hCG administration, number of large follicles, number of oocytes retrieved), were not obtained; therefore, we cannot exclude their impact on OHSS severity. Third, we did not obtain data on live births, which is important for analyzing the effect of OHSS development on pregnancy outcomes. Finally, we did not observe the further evolution of pregnancy; consequently, we cannot compare live births and other pregnancy outcomes between women with moderate and severe OHSS.

## CONCLUSIONS

Estradiol concentration is an associated factor for OHSS in women undergoing COH. In addition, basal serum albumin concentration, together with total protein level and hematocrit, are predictive factors for OHSS severity. It may be speculated that determination of the above parameters after the hCG trigger could help to implement an appropriate strategy to reduce the risk of severe OHSS. Further prospective studies are needed to determine risk factors for OHSS severity.

## References

[r1] Borm G, Mannaerts B. (2000). Treatment with the gonadotrophin-releasing hormone antagonist
ganirelix in women undergoing ovarian stimulation with recombinant follicle
stimulating hormone is effective, safe and convenient: results of a
controlled, randomized, multicentre trial. The European Orgalutran Study
Group. Hum Reprod.

[r2] Corbett S, Shmorgun D, Claman P, REPRODUCTIVE ENDOCRINOLOGY INFERTILITY COMMITTEE, SPECIAL CONTRIBUTOR. (2014). The prevention of ovarian hyperstimulation
syndrome. J Obstet Gynaecol Can.

[r3] Fatemi HM, Popovic-Todorovic B, Humaidan P, Kol S, Banker M, Devroey P, García-Velasco JA. (2014). Severe ovarian hyperstimulation syndrome after
gonadotropin-releasing hormone (GnRH) agonist trigger and “freeze-all”
approach in GnRH antagonist protocol. Fertil Steril.

[r4] Gonadotrophin-releasing hormone antagonists for assisted
reproductive technology (2011). Obstet Gynecol.

[r5] Gurbuz AS, Gode F, Ozcimen N, Isik AZ. (2014). Gonadotrophin-releasing hormone agonist trigger and freeze-all
strategy does not prevent severe ovarian hyperstimulation syndrome: a report
of three cases. Reprod Biomed Online.

[r6] Iorio GG, Carbone L, Conforti A, Rovetto MY, Picarelli S, Cariati F, Strina I, Papanikolaou E, Alviggi C. (2022). Ovarian Hyperstimulation Syndrome after GnRH Agonist Triggering
and Freeze-All Protocol? Never Not, Hardly Ever: A Systematic Review of Case
Reports. Gynecol Obstet Invest.

[r7] McClelland DB. (1990). ABC of transfusion. Human albumin solutions. BMJ.

[r8] Medwin C, Rozen G, Agresta F, Nassar N, Polyakov A. (2023). The ovarian hyperstimulation that truly matters: Admissions,
severity and prevention strategies. Aust N Z J Obstet Gynaecol.

[r9] Nelson SM. (2017). Prevention and management of ovarian hyperstimulation
syndrome. Thromb Res.

[r10] Ovarian Stimulation TEGGO, Bosch E, Broer S, Griesinger G, Grynberg M, Humaidan P, Kolibianakis E, Kunicki M, La Marca A, Lainas G, Le Clef N, Massin N, Mastenbroek S, Polyzos N, Sunkara SK, Timeva T, Töyli M, Urbancsek J, Vermeulen N, Broekmans F. (2020). ESHRE guideline: ovarian stimulation for IVF/ICSI. Hum Reprod Open.

[r11] Patel G, Kashyap A. (2024). Are we really OHSS free?. JBRA Assist Reprod.

[r12] Piróg M, Kacalska-Janssen O, Jach R, Wyroba J, Chrostowski B, Ząbczyk M, Natorska J. (2022). Antagonist Protocol Enhances Coagulation During Controlled
Ovarian Stimulation for IVF. Reprod Sci.

[r13] Piróg MM, Jach R, Undas A. (2016). Thromboprophylaxis in women undergoing gynecological surgery or
assisted reproductive techniques: new advances and
challenges. Ginekol Pol.

[r14] Practice Committee of the American Society for Reproductive
Medicine (2016). Electronic address: ASRM@asrm.org; Practice Committee of the
American Society for Reproductive Medicine. Prevention and treatment of
moderate and severe ovarian hyperstimulation syndrome: a
guideline. Fertil Steril.

[r15] Romito I, Gulino FA, Laganà AS, Vitale SG, Tuscano A, Leanza G, Musmeci G, Leanza V, Rapisarda AM, Palumbo MA. (2017). Renal and Hepatic Functions after A Week of Controlled Ovarian
Hyperstimulation during. Int J Fertil Steril.

[r16] The Royal College of Obstetricians and Gynaecologists (2016). Green-top Guideline No.

[r17] Schon SB, Kelley AS, Jiang C, Xu M, Menke M, Marsh EE. (2022). Emergency department utilization for ovarian hyperstimulation
syndrome. Am J Emerg Med.

[r18] Schouten N, Wang R, Torrance H, Van Tilborg T, Bastu E, Bergh C, D’Hooghe T, Friis Petersen J, Jayaprakasan K, Khalaf Y, Klinkert E, La Marca A, Vuong L, Lapensée L, Lensen S, Magnusson Å, Allegra A, Nyboe Andersen A, Oudshoorn S, Popovic-Todorovic B (2025). Development and validation of a gonadotropin dose selection model
for optimized ovarian stimulation in IVF/ICSI: an individual participant
data meta-analysis. Hum Reprod Update.

[r19] Shah DK, Missmer SA, Correia KF, Ginsburg ES. (2014). Pharmacokinetics of human chorionic gonadotropin injection in
obese and normal-weight women. J Clin Endocrinol Metab.

[r20] Sun B, Ma Y, Li L, Hu L, Wang F, Zhang Y, Dai S, Sun Y. (2020). Associated with Ovarian Hyperstimulation Syndrome (OHSS) Severity
in Women With Polycystic Ovary Syndrome Undergoing IVF/ICSI. Front Endocrinol (Lausanne).

[r21] Tang H, Mourad SM, Wang A, Zhai SD, Hart RJ. (2021). Dopamine agonists for preventing ovarian hyperstimulation
syndrome. Cochrane Database Syst Rev.

[r22] Timmons D, Montrief T, Koyfman A, Long B. (2019). Ovarian hyperstimulation syndrome: A review for emergency
clinicians. Am J Emerg Med.

[r23] Toftager M, Bogstad J, Bryndorf T, Løssl K, Roskær J, Holland T, Prætorius L, Zedeler A, Nilas L, Pinborg A. (2016). Risk of severe ovarian hyperstimulation syndrome in GnRH
antagonist versus GnRH agonist protocol: RCT including 1050 first IVF/ICSI
cycles. Hum Reprod.

[r24] Wang Q, Wan Q, Li T, Wang X, Hu Y, Zhong Z, Pu K, Ding Y, Tang X. (2024). Effect of GnRH agonist trigger with or without low-dose hCG on
reproductive outcomes for PCOS women with freeze-all strategy: a propensity
score matching study. Arch Gynecol Obstet.

[r25] Xiao JS, Su CM, Zeng XT. (2014). Comparisons of GnRH antagonist versus GnRH agonist protocol in
supposed normal ovarian responders undergoing IVF: a systematic review and
meta-analysis. PLoS One.

[r26] Yan Y, Jiang J, Mei J, Shen X, Jiang Y, Xing J, Huang C. (2025). Impact of Elevated Oestrogen Levels on hCG Trigger Day on
Clinical Pregnancy Outcome and OHSS Incidence in Long-Acting GnRHa
Down-Regulated IVF/ICSI-ET Cycles. Int J Womens Health.

[r27] Youssef MA, Al-Inany HG, Evers JL, Aboulghar M. (2011). Intra-venous fluids for the prevention of severe ovarian
hyperstimulation syndrome. Cochrane Database Syst Rev.

[r28] Youssef MA, Mourad S. (2016). Volume expanders for the prevention of ovarian hyperstimulation
syndrome. Cochrane Database Syst Rev.

